# A Geometric Perspective on Information Plane Analysis

**DOI:** 10.3390/e23060711

**Published:** 2021-06-03

**Authors:** Mina Basirat, Bernhard C. Geiger, Peter M. Roth

**Affiliations:** 1 Institute of Computer Graphics and Vision, Graz University of Technology, Inffeldgasse 16/II, 8010 Graz, Austria; basirat@icg.tugraz.at; 2 Know-Center GmbH, Inffeldgasse 13, 8010 Graz, Austria; geiger@ieee.org; 3 International AI Future Lab, Technical University of Munich (TUM), Willy-Messerschmitt-Straße 1, 85521 Taufkirchen, Germany

**Keywords:** information plane analysis, image classification, neural networks, adaptive and fixed binning

## Abstract

Information plane analysis, describing the mutual information between the input and a hidden layer and between a hidden layer and the target over time, has recently been proposed to analyze the training of neural networks. Since the activations of a hidden layer are typically continuous-valued, this mutual information cannot be computed analytically and must thus be estimated, resulting in apparently inconsistent or even contradicting results in the literature. The goal of this paper is to demonstrate how information plane analysis can still be a valuable tool for analyzing neural network training. To this end, we complement the prevailing binning estimator for mutual information with a geometric interpretation. With this geometric interpretation in mind, we evaluate the impact of regularization and interpret phenomena such as underfitting and overfitting. In addition, we investigate neural network learning in the presence of noisy data and noisy labels.

## 1. Introduction

Deep Learning (e.g., [1,2,3,4]) has shown promising performance for many applications including image analysis, speech analysis, or robotics. This progress, however, is mainly the result of more and more sophisticated neural network (NN) architectures with ever-increasing complexity. This makes it increasingly difficult to understand how such NNs work and to interpret or explain their predictions correctly, in particular, if the number of parameters or training data increase. Thus, neither a simple validation of the input–output mapping nor focusing on salient features rather than all possible parameters [5,6,7,8,9,10,11,12,13] are sufficient in practice.

**Figure 1:**
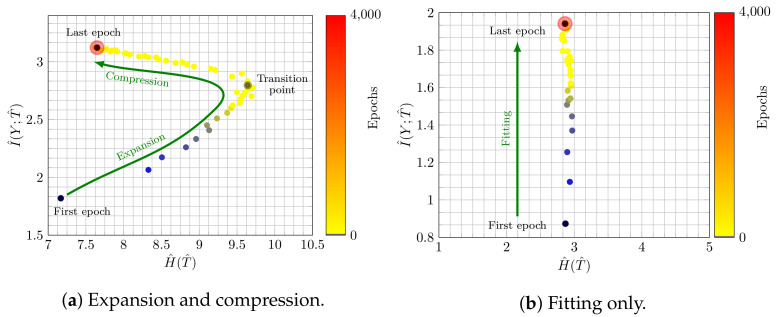
IP for epochs 1–4000: (**a**) fitting occurring simultaneously with an initial expansion phase and a later compression phase; (**b**) only a fitting phase. In contrast to the prevailing literature, we label the axes with $\hat{H}\left(\hat{T}\right)$ and $\hat{I}\left(Y;\hat{T}\right)$ to make the dependence on the estimator more explicit. (See the text for details.) The first epoch is highlighted by a dark color and the size of the last epoch is larger than others.

Thus, research has focused on understanding the inner workings of NNs and investigating, for instance, the learning behavior over time. One prominent example is information plane (IP) analysis [14], which is based on the information bottleneck principle [15]. The key idea is to analyze the plane described by the mutual information I(X;T) between the input *X* and the activation values of a hidden layer *T* and by the mutual information I(Y;T) between *T* and the target variable *Y*, and how these values change from epoch to epoch. Illustrative examples showing the trajectory of mutual information values over time are shown in Figure 1. Even though IPs appear to be an appealing way to analyze learning behaviors of NNs, we face the problem that the literature on IP analysis reports conflicting results, cf. [14,16,17].

This apparent conflict results from the fact that the mutual information can often not be computed analytically. Thus, contradicting results stem from different ways to estimate the mutual information terms I(X;T) and I(Y;T). For instance, mutual information has been approximated via binning, i.e., via discretizing the continuous activation values; the authors have proposed fixed uniform binning [14,18,19], adaptive uniform binning [16], and adaptive nonuniform binning [20]. However, more elaborate estimation schemes have also been used, such as kernel density estimation [16,21], neural estimators based on the Donsker–Varadhan representation of mutual information [22], estimators based on hash functions [23], and kernel-based estimators [24].

This diversity of estimators is unproblematic as they should all converge to the same estimates, e.g., $\hat{I}\left(X;T\right)\approx I\left(X;T\right)$ if the following conditions hold: (a) the required mutual information terms I(X;T) and I(Y;T) are finite, (b) sufficient data are available for their estimation, and (c) the estimators $\hat{I}\left(\cdot ;\cdot \right)$ are adequately parametrized. However, in [25], it was shown that I(X;T) is indeed infinite for deterministic neural networks, concluding that the (finite) estimates $\hat{I}\left(X;T\right)$ depend more on the exact estimation procedure than on the true values. Therefore, the IPs do not exactly depict mutual information, causing the abovementioned ambiguities.

The goal of this paper is to demonstrate that IPs can still be a valuable tool for analyzing NN training if the estimates are interpreted correctly. In particular, similar to recent findings [17,18], we demonstrate that IPs represent geometric rather than information-theoretic phenomena. To this end, we create an IP from the plugin estimates for mutual information between the uniformly discretized activation value $\hat{T}$ and the network input *X* or class label *Y*, respectively. Introducing both fixed and adaptive binning schemes for obtaining $\hat{T}$, we argue that the correct interpretation of $\hat{I}\left(X;\hat{T}\right)$ yields an insight into the geometric compression of the activation *T*, both in absolute (e.g., describing the diameter of the set of all activations of a dataset) and relative (e.g., clustering of activations of a dataset) terms. To allow for a more intuitive interpretation, we additionally show a 2D visualization of latent space.

In summary, the main contributions of the paper are the following:

1. We introduce an interpretation for the estimates of mutual information from a geometric perspective, for both fixed and adaptive uniform binning. We support the interpretation by visualizing the data distribution in the latent space.
2. We show that the effects of regularization and phenomena such as overfitting and underfitting can be well described and interpreted via an IP analysis based on this geometric perspective.
3. Based on the geometric interpretation of IP analyses, we investigate robust classifier learning, in particular, being able to provide an interpretation of the learning behavior in the presence of noisy data and noisy labels.

The rest of the paper is organized as follows: First, in Section 2, we review and discuss the main ideas of IP analysis and introduce and discuss our approach. Then, in Section 3, we apply these findings to provide a thorough evaluation of deep NN learning for image classification tasks. Finally, in Section 4 we summarize and conclude our work.

## 2. Information Planes and Their Geometric Interpretation

Given a labeled training set $D=\{\left(x_{1},y_{1}\right),\cdots ,\left(x_{N},y_{N}\right)\}$, where $x_{i}$ are data points and $y_{i}$ are the corresponding class labels, the goal is to train a deep fully connected feed-forward NN with *L* layers. Assuming that D contains independent samples of a joint distribution $P_{X,Y}$, the data points $x_{i}$ and the class labels $y_{i}$ can be interpreted in the following as random variables *X* and *Y*, respectively.

Let $t_{\ell ,i}$ denote the vector of activation values of the *ℓ*th layer for a data point $x_{i}$. During training, the activation values change from epoch to epoch even for the same data point, i.e., $t_{\ell ,i}\left(n\right)$ is a function of the epoch index *n*. In the following, we suppress this index for the sake of readability. Since the NNs we consider are deterministic, there exists a function $f_{\ell }$ for mapping a feature to this activation vector: $t_{\ell ,i}=f_{\ell }\left(x_{i}\right)$. The activation vectors $\left\{t_{\ell ,1},\cdots ,t_{\ell ,N}\right\}$ can be assumed to be independent realizations of a random variable $T_{\ell }=f_{\ell }\left(X\right)$. For the sake of readability, we write *T* instead of $T_{\ell }$, as the layer index is clear from the context.

In the following, we first discuss the estimation of mutual information for NNs via binning, which is a common approach to construct the IP. Second, we show that binning inherently introduces a geometric perspective that helps to interpret the IP correctly.

### 2.1. Mutual Information Estimation for Neural Network Training

For a pair of discrete random variables, *U* and *V* with joint probability mass function $p_{U,V}$, the mutual information can be readily computed via ([26], Equation (2.28))

(1) $$I\left(U;V\right)=\sum_{u,v}p_{U,V}\left(u,v\right)\log\frac{p_{U,V}\left(u,v\right)}{p_{U}\left(u\right)p_{V}\left(v\right)},$$

where $p_{U}$ and $p_{V}$ are the marginal distributions of *U* and *V*, respectively. In general, we can compute the mutual information if *U* and *V* are both continuous and if the joint probability density function (PDF) $f_{U,V}$ exists and is known ([26], Equation (9.47)).

For NNs, the distributions of the data points *X* and activations *T* are often assumed to be continuous, but the PDFs are not readily available. Thus, even assuming that the joint PDF exists, we require estimators for mutual information that are based on the dataset D, such as kernel density estimators [16] or binning estimators [14,18,19,20]. If the joint PDF exists and if the true value of I(X;T) is finite, then there exist estimators $\hat{I}\left(X;T\right)$ that can be parameterized such that $\hat{I}\left(X;T\right)\approx I\left(X;T\right)$; at least if D is sufficiently large. However, in [25], it was shown that the joint PDF of *X* and *T* does not exist for deterministic NNs. Indeed, since $T=f_{\ell }\left(X\right)$, we have I(X;T)=∞ for continuous input distributions and many practically relevant activation functions ([25], Theorem 1). The estimates of I(X;T) based on a finite dataset are thus inadequate.

To circumvent this problem, we rather focus on the mutual information between *X* or *Y* and a discretized version $\hat{T}$ of the activation *T*. This discretization, which we obtain via binning, ensures that the mutual information terms are finite and, thus, can be estimated reliably. Specifically, rather than estimating I(X;T) and I(Y;T) directly, we estimate $I(X;\hat{T})$ and $I(Y;\hat{T})$, where $\hat{T}$ is obtained by uniformly quantizing (binning) *T*:

(2) $$\hat{T}=\left\lceil \frac{T}{b}\right\rceil ,$$

where *b* is the size of the bin and ⌈·⌉ is the ceiling operator applied to each element of the scaled activation vector. Specifically, we introduce two binning schemes: (a) binning with a fixed bin size of b=0.5 and (b) binning with an adaptive bin size, where for each coordinate of *T*, *b* is one-tenth of the range of activation values of this coordinate over the dataset. In other words, if $t_{\ell ,i}^{\left(j\right)}\left(n\right)$ is the activation value of the *j*th neuron in the *ℓ*th layer for data point *i* at epoch *n*, then

(3) $$b^{\left(j\right)}\left(n\right)=\frac{\max_{i}t_{\ell ,i}^{\left(j\right)}\left(n\right)-\min_{i^{\prime }}t_{\ell ,i^{\prime }}^{\left(j\right)}\left(n\right)}{10}$$

and $T^{\left(j\right)}\left(n\right)=\left\lceil T^{\left(j\right)}\left(n\right)/b^{\left(j\right)}\left(n\right)\right\rceil$ and $T\left(n\right)=(T^{\left(1\right)}\left(n\right),T^{\left(2\right)}\left(n\right),\cdots )$.

Since *T* (and thus $\hat{T}$) is a deterministic function of *X*, we have $I\left(X;\hat{T}\right)=H\left(\hat{T}\right)$ ([26], Equations (2.41) and (2.167)). Moreover, both *Y* and $\hat{T}$ are discrete random variables, and both $H(\hat{T})$ and $I(Y;\hat{T})$ can be estimated using the plugin estimators for entropy and mutual information. Specifically, with ${\hat{t}}_{i}=\left\lfloor t_{i}/b\right\rfloor$, we have

(4a) $$\begin{array}{cc} \; & \hat{H}\left(\hat{T}\right)=-\sum_{t}\frac{\left|\{i:\;{\hat{t}}_{i}=t\}\right|}{N}\log\frac{\left|\{i:\;{\hat{t}}_{i}=t\}\right|}{N} \end{array}$$

(4b) $$\begin{array}{cc} \; & \hat{I}\left(Y;\hat{T}\right)=\sum_{t,y}\frac{\left|\{i:\;{\hat{t}}_{i}=t,y_{i}=y\}\right|}{N}\log\frac{N|\{i:\;{\hat{t}}_{i}=t,y_{i}=y\}|}{|\left\{i:\;{\hat{t}}_{i}=t\right\}\left|\right|\left\{i:\;y_{i}=y\right\}|.} \end{array}$$

These estimators are reasonable if the number of data points for each combination of *t* and *y* in the sums is sufficiently large. However, for many applications, this is rarely the case. Indeed, it has been observed that, especially in convolutional NNs, the vector of activations *T* is so large that $\hat{H}\left(\hat{T}\right)\approx \log\left|D\right|$, i.e., every data point in D falls into a different bin, even if the bin size is large (see, for example, Figure 7 in [18]).

### 2.2. Information Plane Analysis

Assuming that the data allows us to estimate information-theoretic quantities involving the random variables over images, class labels, and activation functions, we can calculate the quantities defined in Equation (4). The authors of [14] proposed to plot these values in a Cartesian coordinate system, yielding the so-called *information plane (IP)* and to analyze how they change throughout training. This is illustrated in Figure 1 for two examples.

From Figure 1a, two phases can been observed, cf. [14]: first, a phase in which both $\hat{H}\left(\hat{T}\right)$ (expansion) and $\hat{I}\left(Y;\hat{T}\right)$ (fitting) increase and, second, a compression phase during which $\hat{H}\left(\hat{T}\right)$ decreases ($\hat{I}\left(Y;\hat{T}\right)$ increases only slightly). The compression phase was interpreted as the hidden layer *T* discarding irrelevant information about the input *X* and was causally connected to generalization. In contrast, Figure 1b shows only fitting as an increase in $\hat{I}\left(Y;\hat{T}\right)$.

### 2.3. Interpretation of IPs Based on Binning Estimators

In Section 2.1, we showed that I(X;T) is infinite in deterministic NNs with continuous inputs and thus escapes estimation. To show that IP analyses as introduced in Section 2.2 are still useful, we build on the observation that the horizontal axis, labeled with $\hat{H}\left(\hat{T}\right)$ in our case, does not describe an information-theoretic compression in the sense of a reduction of I(X;T). Such a reduction would indicate that irrelevant features of *X* are discarded when creating the latent representation of a hidden layer with activations *T*; *T* would become conceptually close to a minimal sufficient statistic. Rather, the current consensus is that $\hat{H}\left(\hat{T}\right)$ is a measure of geometric size and that, thus, compression observed in the IP using such estimators is geometric [17,18]: the quantity $\hat{H}\left(\hat{T}\right)$ is small if the image of the dataset D under the NN function $f_{\ell }$ occupies only a few bins or many bins but with a heavily skewed distribution. In such cases, $f_{\ell }\left(D\right)$ has either a small diameter (relative to a fixed bin size) or is strongly clustered (if the bin size is adapted to the range of activation values.

To improve the intuitive understanding, we consider three cases: (1) All data points are mapped to a small region in feature space that is covered by a single bin. Then, $\hat{T}$ is constant over D and $\hat{H}\left(\hat{T}\right)=0$ (see Figure 6a). (2) All data points are clustered, i.e., data points belonging to one class are mapped to a small region in the feature space, and regions corresponding to different classes are far apart. Furthermore, data points belonging to one class all fall within the same bin, but different classes occupy different bins. Then, $\hat{H}\left(\hat{T}\right)$ is related to the logarithm of the number of classes. (3) The data points are spread over the feature space so that every bin contains at most one data point. Then, we have $\hat{H}\left(\hat{T}\right)=\log\left|D\right|$. This can occur either if the latent space is very high-dimensional as in convolutional NNs or if the bin size *b* chosen is too small.

In all three cases, $\hat{H}\left(\hat{T}\right)$ is a measure of the geometric “size” of the image $f_{\ell }\left(D\right)$ in the latent space, where “size” has to be interpreted probabilistically and is measured relative to the bin size *b*: $f_{\ell }\left(D\right)$ is “small” in this sense if some majority of its elements are covered by only few bins. While fixed binning measures the geometric size with an absolute scale, adaptive binning measures the geometric size with a scale relative to the image of the dataset D under *f*, i.e., relative to the absolute scale of the latent space. Thus, a simple scaling of *T*, for instance by scaling all weights in a NN with ReLU activation functions, affects $\hat{H}\left(\hat{T}\right)$ when $\hat{T}$ is obtained by fixed binning but not for adaptive binning.

## 3. Analyzing NN Training via Information Plane Analysis

The goal of our experiments is to demonstrate that IP analysis—if interpreted correctly—can be a useful tool to analyze and interpret NN learning. To this end, we address different problems and tasks: (a) illustrating the impact of regularization; (b) analyzing phenomena such as overfitting and underfitting; and (c) demonstrating the generality of the approach, by applying it to analyze robust learning. For the first two tasks, we run experiments on the well-known MNIST dataset [27]. For the third task, we run experiments on two different benchmark datasets, namely *Brightness MNIST* [28] (noisy data) and *Noisy MNIST* (noisy labels) [29].

For our analysis, we show the mutual information trajectories for both binning approaches, fixed binning (FB) and adaptive binning (AB). For this purpose, after each training epoch, the activation values of the hidden layers evaluated on the test set are saved and the mutual information is computed. For training, we applied an Adam optimizer and used *ReLU* as an activation function, unless noted otherwise. To reduce the influence of random initialization and the inherent randomness of the Adam optimizer, all experiments were run three times for 4000 epochs, respectively.

To visually validate the claim that the IP displays geometric effects, we decided to use a bottleneck architecture with a two-dimensional layer. This allows us to visualize the data set in latent space without having to resort to projection or dimensionality reduction methods such as t-SNE. Even though we show the IP trajectories for all layers, our discussion mainly focuses on the trajectory corresponding to this two-dimensional layer. The findings, however, are more general and hold for different layer sizes and architectures.

### 3.1. Impact of Regularization

**Figure 2:**
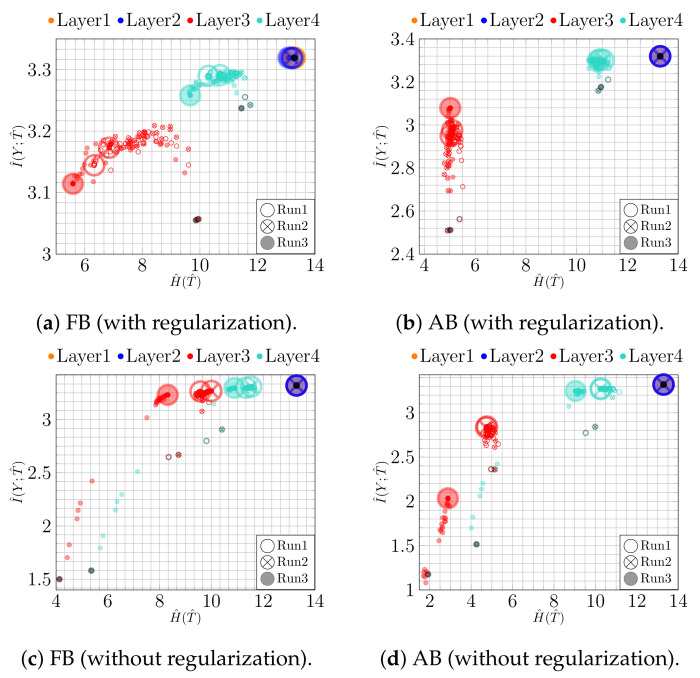
IPs for MNIST (*bottleneck model*): (**a**) fixed and (**b**) adaptive binning with regularization; (**c**) fixed and (**d**) adaptive binning without regularization.

First, we analyze the impact of regularization when training a NN. For this purpose, we trained a bottleneck network (*100-100-2-100*) with and without $l_{2}$ regularization (λ=0.0003, found by grid search). The thus obtained results for both binning approaches are shown in Figure 2. To make the temporal character of the trajectories more apparent, the first and the last epoch are highlighted by a black point and a large circle, respectively.

**Figure 3:**
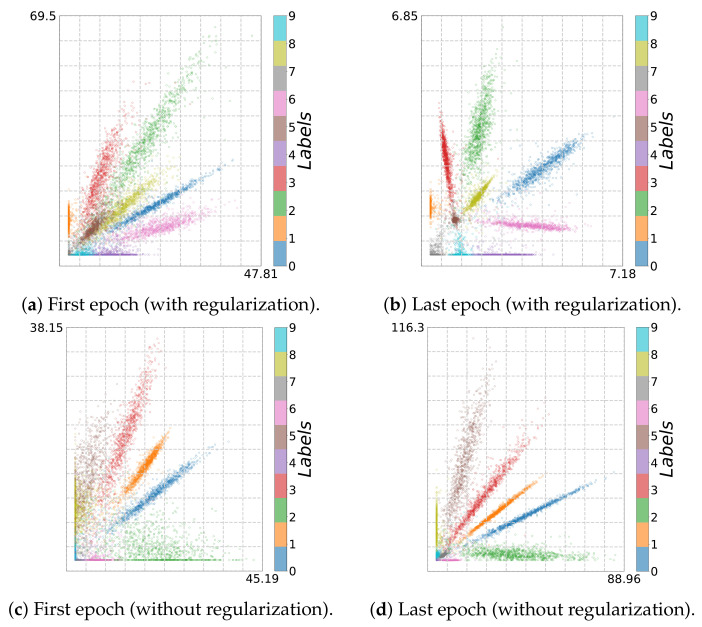
Two-dimensional visualization for MNIST (*bottleneck model*, first and last epoch): (**a**,**b**) with and (**c**,**d**) without regularization.

Using adaptive binning (see Figure 2b), we recognize a fitting phase, i.e., $\hat{I}\left(Y;\hat{T}\right)$ increases over time, indicating a growth in the class separability. In addition, using fixed binning (see Figure 2a), we can recognize a geometric compression with an absolute scale for $\hat{H}\left(\hat{T}\right)$ from the first to the last epoch for the last two layers. Indeed, using an $l_{2}$ regularization (weight decay) reduces the overfitting tendency by keeping the values of the weights small. Consequently, the small weights reduce the absolute scale of the data in latent space. Indeed, as can be seen in Figure 3a,b, where we plot the two-dimensional latent space, the absolute scale reduces from approximately 47×69 to approximately 7×7 during training.

In contrast, as we show in Figure 2c,d, these effects appear not be present without $l_{2}$ regularization. Moreover, in this case, the picture conveyed by the IP is slightly less consistent. For instance, Run 1 and Run 2 show neither a compression nor a fitting phase for fixed binning, as can be seen in Figure 2c. Rather, the latent representation seems to expand throughout training, which is caused by increasing NN weights. This is also illustrated in Figure 3c,d, from which we can see that the absolute scale increases from approximately 45×36 to approximately 88×116. In contrast, for Run 3, we can see mainly an upward trend (only fitting) for $\hat{I}\left(Y;\hat{T}\right)$.

**Figure 4:**
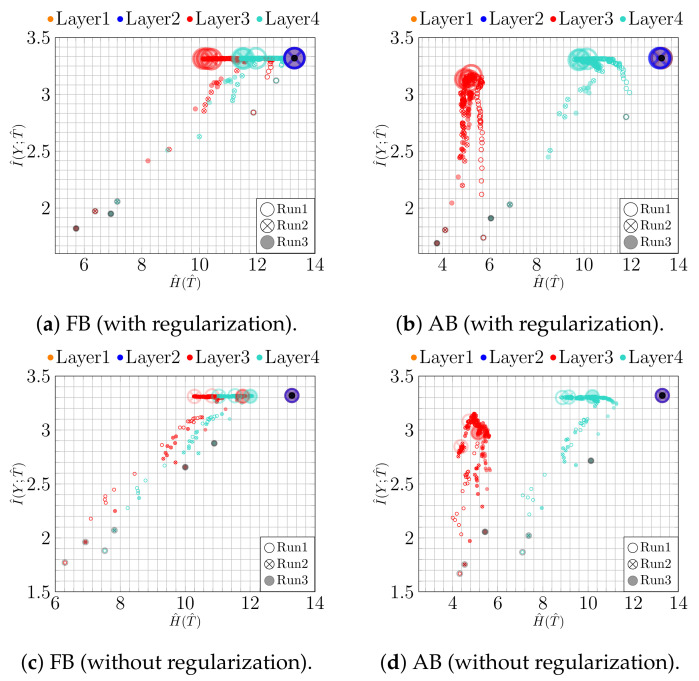
IPs for MNIST (*CNN model*): (**a**) fixed and (**b**) adaptive binning with regularization; (**c**) fixed and (**d**) adaptive binning without regularization.

**Figure 5:**
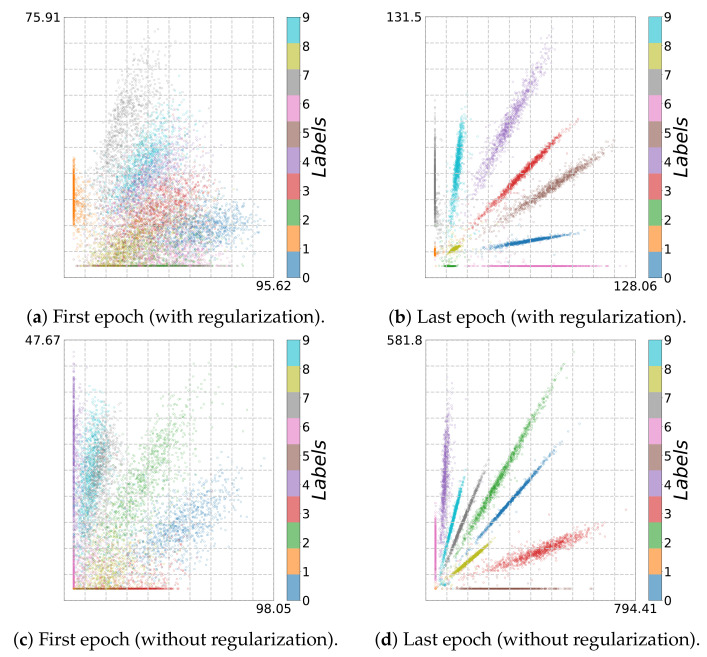
Two-dimensional visualization for MNIST (*CNN model*, first and last epoch): (**a**,**b**) with and (**c**,**d**) without regularization.

We additionally run the same experiment using a convolutional NN (CNN). The CNN consists of four convolutional, two max-pooling, and four fully connected (100-100-2-100) layers. The results for the IP analysis on the fully connected layers are shown in Figure 4. In Figure 4a,c, we can see an expansion phase in fixed binning both with and without regularization. Moreover, the regularization results in a consistent fitting phase and slightly larger values of $\hat{I}\left(Y;\hat{T}\right)$ for adaptive binning at the last epoch, cf. Figure 4b. This can be traced back to better class separability, as seen in Figure 5b. Due to the simplicity of the task, the effect of overfitting on classification accuracy is mild: the full connected NN achieves 96.62% with and 96.42% without regularization, while the CNN achieves 98.90% with and 98.60% without regularization. The corresponding IPs, however, display a qualitatively different behavior, indicating that similar accuracies were achieved along different training paths.

### 3.2. Underfitting Models

**Figure 6:**
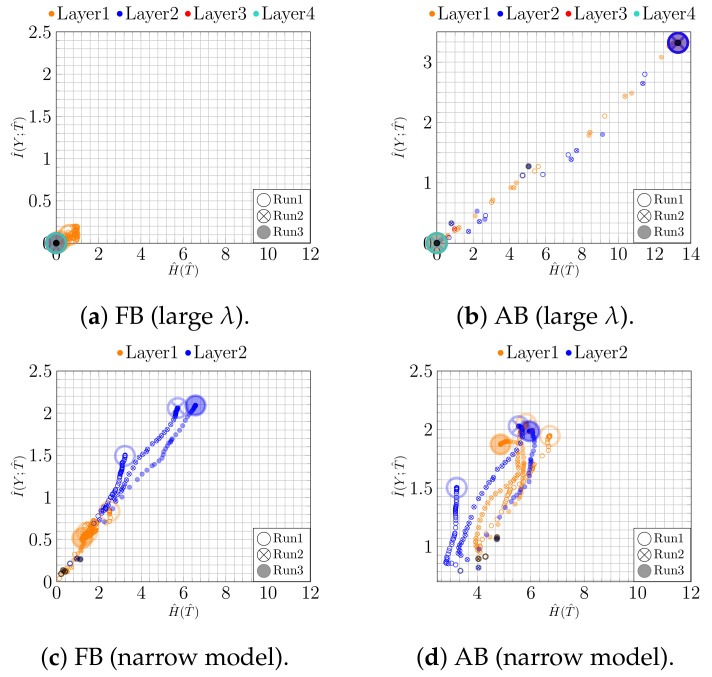
IPs for underfitting model for MNIST: (**a**) fixed and (**b**) adaptive binning for too strong regularization rate (λ) for bottleneck model; (**c**,**d**) fixed and adaptive binning for the narrow model..

The next scenario we consider is underfitting, preventing the model from learning sufficient information from the training data. In this section, we induce underfitting by using (a) too strong regularization (λ=0.2) and (b) a suboptimal network architecture (two layers with three hidden neurons each). The resulting IPs are displayed in Figure 6.

In the first case, using strong regularization, we achieve an accuracy of approximately 11%. Here, all data points are mapped to a small region in feature space that is covered by a single bin. In this case, for fixed binning, $\hat{T}$ is constant over D and $\hat{H}\left(\hat{T}\right)=0$, which is reflected in the IP (see Figure 6a). In addition, the adaptive binning in Figure 6b also shows the same behavior for the last two hidden layers, i.e., $\hat{H}\left(\hat{T}\right)=0$. In the second case, using a too narrow model, we finally obtain an accuracy of approximately 62%, resulting from a slightly different learning behavior. As can be seen from Figure 6c, $\hat{H}\left(\hat{T}\right)$ is small, especially for Layer 1, which means that few bins are overpopulated, whereas others are empty.

Indeed, in both cases, the NN cannot extract relevant and required information from the input to fit the target outputs (*Y*). Therefore, we have $\hat{I}\left(Y;\hat{T}\right)\leq 2$ (see Figure 6), which is lower than log(10)≈3.32 (if all ten classes from MNIST fall into different bins), indicating a weak class separability.

### 3.3. Overfitting Models

The next scenario we consider is overfitting, which can be described as learning a model that fits the training data very well but that does not generalize to unseen data. To demonstrate this in terms of IP analysis, we train a network on MNIST with two hidden layers with 10 units in each layer; in this case, using *tanh* as an activation function. To encourage overfitting, we did not use any regularization.

**Figure 7:**
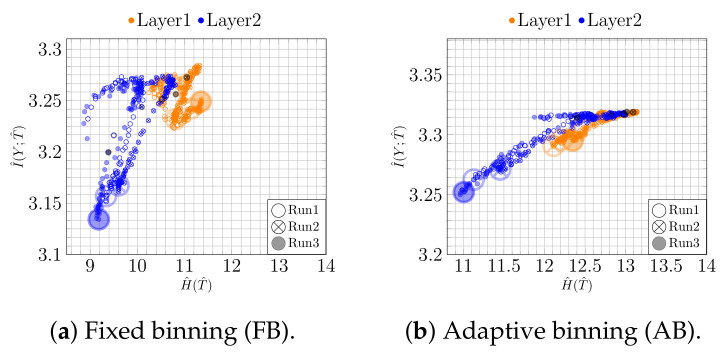
IPs for overfitting model for MNIST: (**a**) fixed and (**b**) adaptive binning.

In contrast to underfitting, which affects the entire IP, overfitting on clean labels can mainly be seen on the vertical axis of the IP. In fact, for an overfitting model, $\hat{I}\left(Y;\hat{T}\right)$ increases at the beginning of the training but decreases again later on. This can be seen in Figure 7.

**Figure 8:**
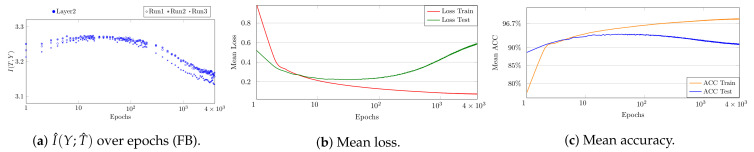
Overfitting model for MNIST: (**a**) the value of $\hat{I}\left(Y;\hat{T}\right)$ over time is related to (**b**) the loss and (**c**) the accuracy.

To further illustrate this effect, in Figure 8a, we plot $\hat{I}\left(Y;\hat{T}\right)$ for Layer 2 over time for fixed binning, first increasing to 3.26 and then decreasing to 3.14 (averaged over three runs). Indeed, this trend (having a peak around epoch 75) is directly related to the learning behavior and the accuracy of the model. To make this more apparent, we compare the plot of $\hat{I}\left(Y;\hat{T}\right)$ to the mean test loss and the mean test accuracy in Figure 8b and Figure 8c respectively. It can be seen that the mean accuracy initially increases up to 93% and then drops (starting around epoch 75) to 90% at the end of the training. The same trend, an initial reduction and subsequent growth, can also be recognized from the loss curve. This indicates that the information covered by IP analysis is directly related to well-known learning characteristics.

### 3.4. Learning from Noisy Data

We next investigate the effect of corrupted input data on the IP. To this end, we run experiments on the *Brightness MNIST* dataset [28], a modified version of MNIST, where the illumination of the images increased. In this way, the contrast of the images decreased and, thus, the classes are pushed closer together in the image space. For evaluation purposes, we train both a *100-100-2-100 bottleneck model* and a *convolutional model* as described before and analyze the learning behavior by using both binning approaches.

**Figure 9:**
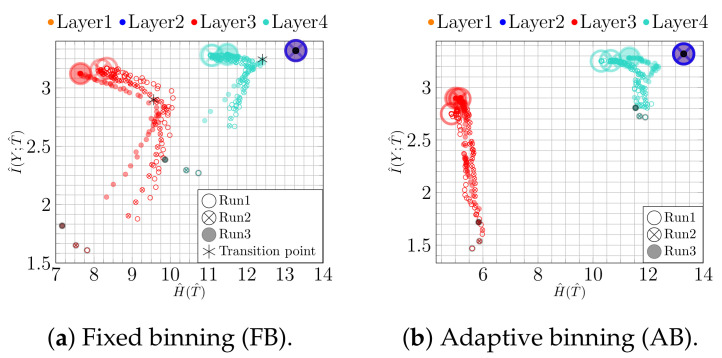
IPs for Brightness MNIST (*bottleneck model*): (**a**) fixed and (**b**) adaptive binning.

**Figure 10:**
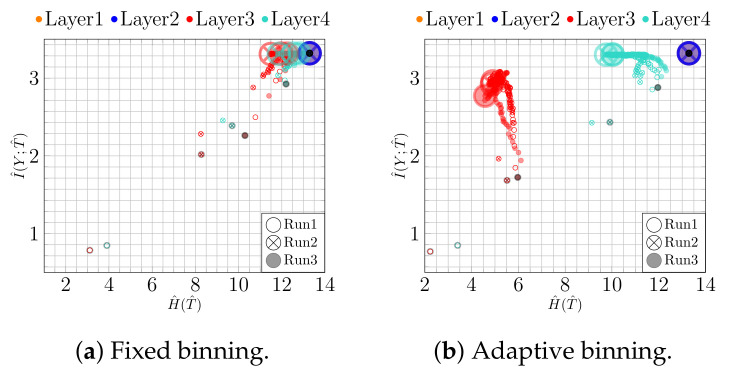
IPs for Brightness MNIST (*CNN model*): (**a**) fixed and (**b**) adaptive binning.

**Figure 11:**
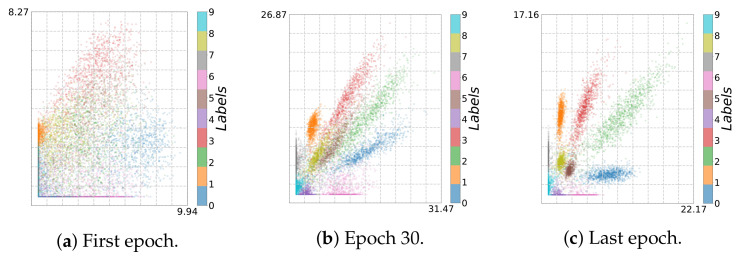
Two-dimensional visualization for Brightness MNIST (*bottleneck model*): (**a**) first and (**c**) last epoch, and (**b**) Epoch 30, describing a transition point.

**Figure 12:**
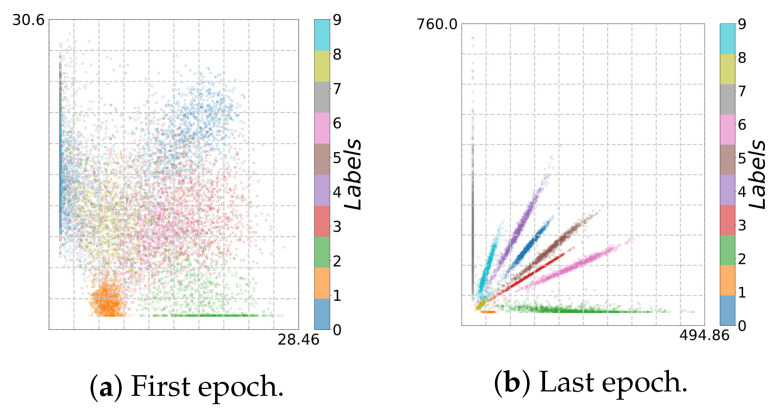
Two-dimensional visualization for *Brightness* MNIST (*CNN model*): (**a**) first and (**b**) last epoch.

Indeed, we finally obtain an accuracy of 95.25% and 98.51% for the bottleneck model and the convolution model, respectively, which is comparable to the results on MNIST using same models. However, the IP analysis shown in Figure 9 and Figure 10 reveal that the learning behavior is different. As can be seen from Figure 11a and Figure 12a, due to reduced contrast in the images, the classes are mapped to highly overlapping regions; this is not the case for the original MNIST dataset (cf. Figure 3c and Figure 5c).

To learn successfully, during NN training, the data points in the latent space have to be pushed apart according to their class label. In this way, we can recognize a fitting phase (increasing $\hat{I}\left(Y;\hat{T}\right)$) for adaptive binning (see Figure 9b and Figure 10b) and an expansion phase for fixed binning (see Figure 9a and Figure 10a). Simultaneously, the data points are pushed apart and occupy a larger volume in latent space (increased from 10×8 to 31×27 and from 30×28 to 769×434), as can be seen in Figure 11 and Figure 12.

For the bottleneck model, after epoch 30 (transition point, see Figure 11b), a compression phase emerges for fixed binning, and the clusters are tightened and separated from each other. For adaptive binning (see Figure 9b and Figure 10b), both models share the same trend: a fitting phase along with a compression phase for the bottleneck layer. Moreover, for the last layer (Layer 4), an expansion phase and subsequently a compression phase can be recognized. Since the IPs for MNIST show a slightly different qualitative behavior, this indicates that the IP displays effects both caused by architectural choices and the selected data set.

### 3.5. Learning from Noisy Labels

For many practical applications, we face the problem of noisy and ambiguous labels in the training data (see, e.g., [30]). Thus, there has been a huge interest in studying the dynamics of NN learning from noisy labels [31,32,33,34,35,36], reaching the consensus that NNs first learn the training data for clean labels and subsequently memorize data for the noisy labels.

We investigate this scenario in the IP using a *100-100-2-100 bottleneck model*. In addition, we evaluate rectifier family activation functions, namely *ReLU* and *Leaky ReLU* (with two different slopes: α=0.01 and α=0.3) and double saturated activation functions, e.g., *Tanh* for noisy labels. For that purpose, similar to [29,37], we apply the idea of symmetric label noise and replace the true label with a label from other classes for 40% of the training samples of MNIST. The thus obtained results for clean and noisy labels are summarized in Table 1.

**Figure 13:**
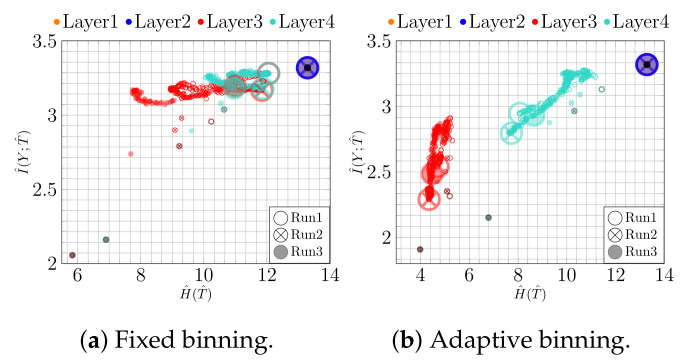
IPs for *Noisy MNIST* using *ReLU*: (**a**) fixed binning and (**b**) adaptive binning.

**Figure 14:**
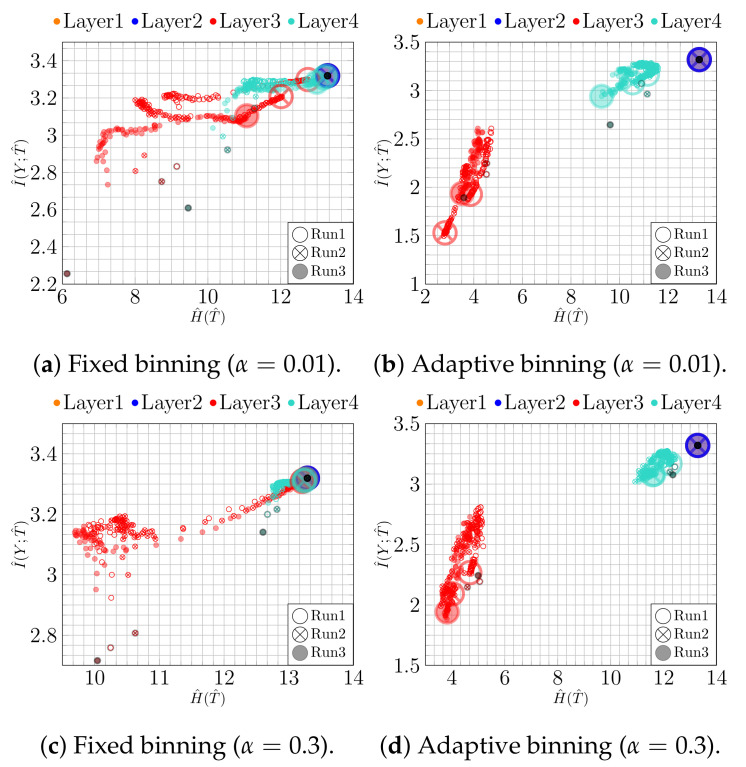
IPs for *Noisy MNIST* using *Leaky ReLU*: (**a**) fixed binning and (**b**) adaptive binning with (α=0.01); (**c**) fixed binning and (**d**) adaptive binning with (α=0.03).

At the beginning of the training process, the weights are randomly initialized close to zero. Therefore, the activation values of the rectified activation functions are small. When training starts, they deviate from the small value and start to increase. Thus, functions of the rectified unit family show an expansion in fixed binning in which $\hat{H}\left(\hat{T}\right)$ increases over time, which can be seen from Figure 13a and Figure 14a,c.

**Figure 15:**
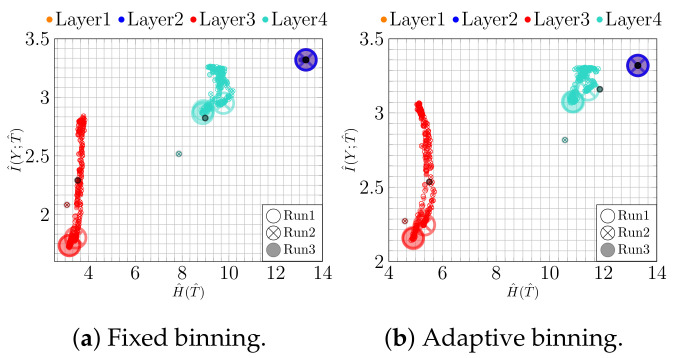
IPs for *Noisy MNIST* using *Tanh*: (**a**) fixed binning and (**b**) adaptive binning.

**Figure 16:**
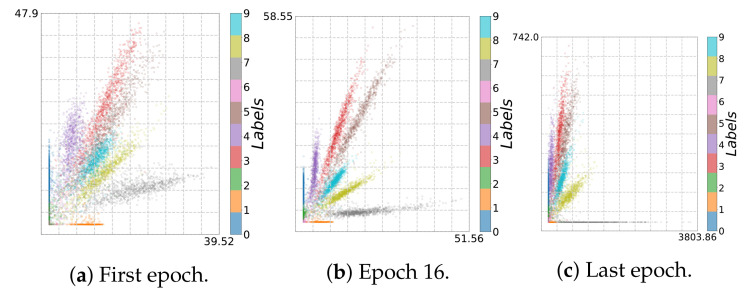
Two-dimensional visualization for *Noisy MNIST* using *ReLU*: (**a**) first epoch, (**b**) Epoch 16, and (**c**) last epoch.

**Figure 17:**
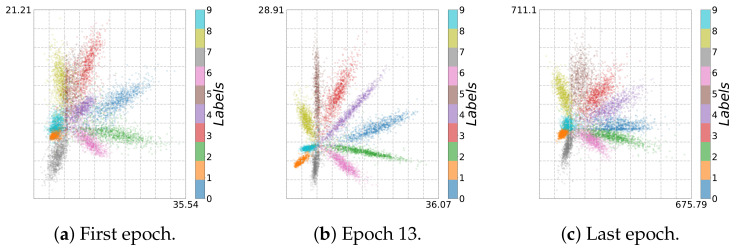
Two-dimensional visualization of *Noisy MNIST* using *Leaky ReLU* with α=0.03: (**a**) first epoch, (**b**) Epoch 13, and (**c**) last epoch.

**Figure 18:**
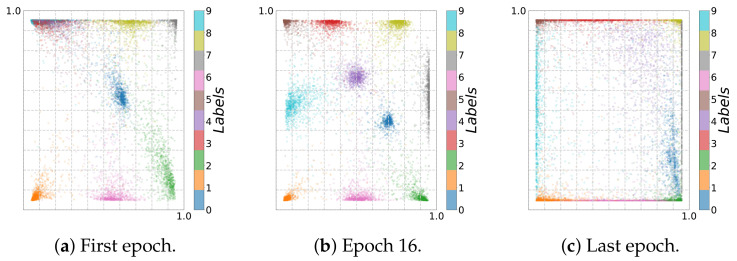
Two-dimensional visualization of *Noisy MNIST* using *Tanh*: (**a**) first epoch, (**b**) Epoch 16, and (**c**) last epoch.

In contrast, the saturation regions of double saturated activation functions restrict the activation values, and we cannot see an expansion using fixed binning (see Figure 15a). This behavior is also reflected in the 2D visualization of the bottleneck layer for rectifying activation functions (see Figure 16c and Figure 17c) by increasing the absolute scale. However, the absolute scale is bounded in the range [−1,1] for *Tanh* (see Figure 18).

**Figure 19:**
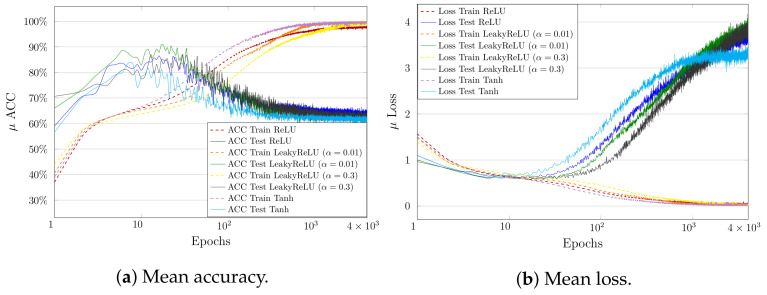
Mean accuracy (**a**) and mean loss (**b**) over time for Noisy MNIST training for all activation functions (averaged over three runs).

In general, when training from noisy labels, the model is first fit to the clean labels and then starts to memorize noisy labels (overfitting). In the fitting phase, $\hat{I}\left(Y;\hat{T}\right)$ increases, which can be seen in the adaptive binning illustrated in Figure 13b, Figure 14b,d and Figure 15b). However, in the memorization phase, $\hat{I}\left(Y;\hat{T}\right)$ decreases. At the same time, the accuracy also decreases and loss increases, as can be seen in Figure 19a and Figure 19b respectively. These two phases indicate first a growth (see Figure 16b, Figure 17b and Figure 18b) and then a reduction of class separability in the 2D visualization (see Figure 16c, Figure 17c and Figure 18c).

In particular, for Epoch 13 (see Figure 17b) and Epoch 16 (see Figure 16b and Figure 18b), we can see the transition between fitting clean labels and fitting noisy ones [29]. This seems to be independent of the activation function used. On the other hand, since *ReLU* maps all negative activation values to zero, we can see a geometric compression (tighter clustering) in this case, especially for the last layer along with decrease in $\hat{I}\left(X;\hat{T}\right)$ in adaptive binning.

## 4. Discussion and Conclusions

The main idea of IP analysis is to analyze the plane described by the mutual information I(X;T) between the input *X* and the activation values of a hidden layer *T* and by the mutual information I(Y;T) between *T* and the output variable *Y* over time. However, as the mutual information cannot be computed analytically, different estimation approaches are used, which leads to inconsistent results and contradicting interpretations of the IPs.

To overcome these issues, as first contribution, we demonstrated that the IP represents geometric rather than information-theoretic effects. To this end, we take advantage of two different binning estimators based on fixed and adaptive binning, requiring different geometric interpretations and thus giving us different views on the geometric compression of the activation *T*. For our experimental results, we used a bottleneck architecture (a two-dimensional layer), which allows us to directly relate the information covered by IPs to the geometric structure of the latent space. Additionally, showing the two-dimensional latent space supports our findings; however, the application of IP analysis is not limited to this type of architecture. To this end, we also showed results using different architectures, demonstrating that—if interpreted correctly—IP analysis can be a valuable tool to analyze neural network training.

Based on these findings, as a second contribution, we analyzed different scenarios for NN training. First, we evaluated and interpreted the impact of regularization and phenomena such as underfitting and overfitting using the well-known MNIST dataset. We showed that the effects of $l_{2}$ regularization, which aims to minimize the magnitude of weights, can be seen both in the IP and the two-dimensional visualization of the latent space. Furthermore, we were able to visualize and interpret over- and underfitting problems for specific setups using IPs. In addition, we also considered practical relevant problems, namely learning from noisy samples and noisy labels. For the first problem, we could show that, despite achieving similar classification performance, the learning behavior is different. For the second problem, we evaluated different activation functions and provided evidence that rectifying activations show an expansion phase corresponding to the memorization of noisy labels. Such an expansion phase is missing for double saturated activation functions, despite them memorizing the noisy labels as well.

In this way, we demonstrated that IPs can be a valuable tool to analyze NN training. However, the mutual information estimators must be adequately designed and their estimates must be interpreted correctly. In particular, we showed that such an interpretation must—at least for binning estimators—take into account geometric aspects. Building on these findings, we will further investigate learning from noisy data and noisy labels. In particular, we are interested in the impact of using different non-linearities for this type of application scenario. Thus, the goal would be to improve the architectural design of deep neural networks when dealing with ambiguous or unreliable data.

## Figures and Tables

**Table 1:** Mean accuracy for MNIST with noisy and clean labels. The best result is in **boldface**, the runner up in *italic*.

Dataset	*Tanh*	*ReLU*	*Leaky ReLU* (α=0.01)	*Leaky ReLU* (α=0.3)
MNIST (Noisy label)	60.95%	62.47%	*62.52%*	**62.78%**
MNIST (Clean label)	96.47%	*96.42%*	95.81%	**96.93%**

## Data Availability

No new data were created or analyzed in this study. Data sharing is not applicable to this article.

## References

[1] Goodfellow I., Bengio Y., Courville A. (2016). Deep Learning.

[2] LeCun Y., Bengio Y., Hinton G. (2015). Deep learning. Nature.

[3] Krizhevsky A., Sutskever I., Hinton G.E. Imagenet Classification with Deep Convolutional Neural Networks. Proceedings of the Neural Information Processing Systems 2012.

[4] Schmidhuber J. (2015). Deep learning in neural networks: An overview. Neural Netw..

[5] Erhan D., Bengio Y., Courville A., Vincent P. (2009). Visualizing Higher-Layer Features of a Deep Network.

[6] Zeiler M.D., Fergus R. (2013). Visualizing and Understanding Convolutional Networks. arXiv.

[7] Simonyan K., Vedaldi A., Zisserman A. (2013). Deep Inside Convolutional Networks: Visualising Image Classification Models and Saliency Maps. arXiv.

[8] Yosinski J., Clune J., Nguyen A., Fuchs T., Lipson H. (2015). Understanding Neural Networks Through Deep Visualization. arXiv.

[9] Nguyen A., Yosinski J., Clune J. (2016). Multifaceted Feature Visualization: Uncovering the Different Types of Features Learned By Each Neuron in Deep Neural Networks. arXiv.

[10] Ribeiro M.T., Singh S., Guestrin C. (2016). “Why Should I Trust You?”: Explaining the Predictions of Any Classifier. arXiv.

[11] Zhou B., Khosla A., Lapedriza A., Oliva A., Torralba A. Learning Deep Features for Discriminative Localization. Proceedings of the 2016 IEEE Conference on Computer Vision and Pattern Recognition (CVPR).

[12] Selvaraju R.R., Cogswell M., Das A., Vedantam R., Parikh D., Batra D. Grad-CAM: Visual Explanations from Deep Networks via Gradient-Based Localization. Proceedings of the 2017 IEEE International Conference on Computer Vision (ICCV).

[13] Zintgraf L.M., Cohen T.S., Adel T., Welling M. (2017). Visualizing Deep Neural Network Decisions: Prediction Difference Analysis. arXiv.

[14] Shwartz-Ziv R., Tishby N. (2017). Opening the Black Box of Deep Neural Networks via Information. arXiv.

[15] Tishby N., Pereira F.C., Bialek W. The Information Bottleneck Method. Proceedings of the Allerton Conference on Communication, Control, and Computing.

[16] Saxe A.M., Bansal Y., Dapello J., Advani M., Kolchinsky A., Tracey B.D., Cox D.D. (2000). On the Information Bottleneck Theory of Deep Learning. arXiv.

[17] Geiger B.C. (2020). On Information Plane Analyses of Neural Network Classifiers—A Review. arXiv.

[18] Goldfeld Z., Van Den Berg E., Greenewald K., Melnyk I., Nguyen N., Kingsbury B., Polyanskiy Y. (2018). Estimating Information Flow in Deep Neural Networks. arXiv.

[19] Schiemer M., Ye J. Revisiting the Information Plane. https://openreview.net/forum?id=Hyljn1SFwr.

[20] Chelombiev I., Houghton C.J., O’Donnel C. (2019). Adaptive Estimators show Information Compression in Deep Neural Networks. arXiv.

[21] Fang H., Wang V., Yamaguchi M. (2018). Dissecting Deep Learning Networks—Visualizing Mutual Information. Entropy.

[22] Elad A., Haviv D., Blau Y., Michaeli T. Direct Validation of the Information Bottleneck Principle for Deep Nets. Proceedings of the 2019 IEEE/CVF International Conference on Computer Vision Workshop (ICCVW).

[23] Noshad M., Zeng Y., Hero A.O. Scalable Mutual Information Estimation Using Dependence Graphs. Proceedings of the 2019 IEEE International Conference on Acoustics, Speech and Signal Processing (ICASSP).

[24] Wickstrøm K., Løkse S., Kampffmeyer M., Yu S., Principe J., Jenssen R. (2019). Information Plane Analysis of Deep Neural Networks via Matrix-Based Rényi’s Entropy and Tensor Kernels. arXiv.

[25] Amjad R.A., Geiger B.C. (2020). Learning Representations for Neural Network-Based Classification Using the Information Bottleneck Principle. IEEE Trans. Pattern Anal. Mach. Intell..

[26] Cover T.M., Thomas J.A. (1991). Elements of Information Theory.

[27] LeCun Y., Bottou L., Bengio Y., Haffner P. (1998). Gradient-based learning applied to document recognition. Proc. IEEE.

[28] Mu N., Gilmer J. (2019). MNIST-C: A Robustness Benchmark for Computer Vision. arXiv.

[29] Arpit D., Jastrzebski S., Ballas N., Krueger D., Bengio E., Kanwal M.S., Maharaj T., Fischer A., Courville A.C., Bengio Y. A Closer Look at Memorization in Deep Networks. Proceedings of the 34th International Conference on Machine Learning.

[30] Northcutt C.G., Athalye A., Mueller J. (2021). Pervasive Label Errors in Test Sets Destabilize Machine Learning Benchmarks. arXiv.

[31] Frenay B., Verleysen M. (2014). Classification in the Presence of Label Noise: A Survey. IEEE Trans. Neural Networks Learn. Syst..

[32] Zhang C., Bengio S., Hardt M., Recht B., Vinyals O. (2017). Understanding deep learning requires rethinking generalization. arXiv.

[33] Jiang L., Zhou Z., Leung T., Li L.J., Fei-Fei L. MentorNet: Learning Data-Driven Curriculum for Very Deep Neural Networks on Corrupted Labels. Proceedings of the 35th International Conference on Machine Learning.

[34] Patrini G., Rozza A., Menon A., Nock R., Qu L. Making Deep Neural Networks Robust to Label Noise: A Loss Correction Approach. Proceedings of the 2017 IEEE Conference on Computer Vision and Pattern Recognition (CVPR).

[35] Natarajan N., Dhillon I.S., Ravikumar P.K., Tewari A. Learning with Noisy Labels. Proceedings of the Neural Information Processing Systems.

[36] Zhang Z., Zhang H., Arik S.O., Lee H., Pfister T. Distilling Effective Supervision From Severe Label Noise. Proceedings of the IEEE/CVF Conference on Computer Vision and Pattern Recognition (CVPR).

[37] Ma X., Wang Y., Houle M.E., Zhou S., Erfani S.M., Xia S., Wijewickrema S.N.R., Bailey J. Dimensionality-Driven Learning with Noisy Labels. Proceedings of the 35th International Conference on Machine Learning.

